# Is the Prognostic Nutritional Index a Novel Prognostic Factor in Patients With Unresectable/Metastatic Gallbladder and Cholangiocarcinoma Receiving Chemotherapy?

**DOI:** 10.7759/cureus.65003

**Published:** 2024-07-20

**Authors:** İlknur Deliktaş Onur, Hatice Gülgün Fırat, Elif Sertesen Çamöz, Fatih Yildiz

**Affiliations:** 1 Department of Medical Oncology, University of Health Sciences, Dr. Abdurrahman Yurtaslan, Ankara Oncology Education and Research Hospital, Ankara, TUR; 2 Department of Internal Medicine, University of Health Sciences, Dr. Abdurrahman Yurtaslan, Ankara Oncology Education and Research Hospital, Ankara, TUR

**Keywords:** systemic chemotherapy (stc), biliary tract tumor, prognostic nutritional index, overall survival (os), cancer gallbladder

## Abstract

Gallbladder and biliary tract tumors are rare but highly fatal cancers. In patients diagnosed with unresectable/metastatic gallbladder cancer and cholangiocarcinomas, systemic chemotherapy is recommended if the patient's performance is good. Randomized studies on this subject are limited, and there is no standard treatment choice. The prognostic nutritional index (PNI) is a measurement calculated using albumin and absolute lymphocyte value, reflecting the immunological and nutritional status of the cancer patient. The aim of our study is to evaluate the prognostic effectiveness of PNI in unresectable/metastatic gallbladder and biliary tract cancers. The PNI was calculated using albumin and lymphocyte values at the time of diagnosis (10 x albumin g/dL + 0.005 x total lymphocyte/mm^3^). The relationship between PNI and overall survival (OS) and progression-free survival was examined. The prognostic nutritional index means of the patients included in the study was 44.8 (95% CI: 42.9-46.7), and the median was 44.77 (minimum: 22, maximum: 61.4). Receiver operating characteristic (ROC) analysis demonstrated a statistically significant prediction of patients' OS when the prognostic nutritional index was < 44 (AUC: 0.715, sensitivity: 54.8%, specificity: 33.3%; p=0.08). We evaluated the prognostic effectiveness of PNI in the subgroup of patients who could receive chemotherapy. In patients receiving chemotherapy, median survival was found to be 8.93 months in the PNI < 44 groups, while median survival was found to be 12.58 months in the PNI ≥ 44 group. The difference between both groups was statistically significant (p = 0.01). In univariate analysis, the Eastern Cooperative Oncology Group (ECOG) performance status, cancer antigen 19.9 (Ca 19.9), and PNI were statistically significant variables in predicting OS (p < 0.05). In multivariate analysis, the ECOG performance status, cancer antigen 19.9 (Ca 19.9), and PNI were found to be independent factors in predicting OS (p < 0.05). We believe that PNI can be used as a marker to assist the clinician in evaluating the prognosis of patients in the clinic and predicting treatment tolerance.

## Introduction

Gallbladder and biliary tract tumors are rare but highly fatal cancers. The majority of gallbladder cancers are found incidentally in patients being investigated for cholelithiasis [1]. Poor prognosis is thought to be related to the advanced stage at diagnosis, depending on both the anatomical position of the gallbladder and the non-specificity of symptoms [2]. Many risk factors have been identified in gallbladder tumors, and most of the risk factors are related to chronic inflammation [3]. Among these risk factors are cholelithiasis, porcelain gallbladder, gallbladder polyps, primary sclerosing cholangitis, and salmonella infection [4]. The primary treatment of gallbladder tumors is surgery. Adjuvant therapy is recommended in early-stage completely resected muscle-invasive (≥T1b) or lymph node-positive or surgical margin-positive patients. Adjuvant chemoradiotherapy is recommended in patients with positive surgical margins or lymph node metastases and capecitabine monotherapy for six months or capecitabine plus gemcitabine in patients with negative surgical margins [5]. Depending on the patient's tolerance, gemcitabine plus cisplatin, gemcitabine plus oxaliplatin, or gemcitabine can be used as a single agent. Cholangiocarcinomas are rare malignancies originating from the epithelial cells of intrahepatic and extrahepatic bile ducts [6]. Cholangiocarcinoma is an aggressive tumor that can metastasize beyond the bile ducts to other intrahepatic regions, the peritoneum, and distant extrahepatic organs. Risk factors for cholangiocarcinoma are as follows: primary sclerosing cholangitis, fibropolycystic liver disease, and chronic intrahepatic stone disease (hepatolithiasis, also called recurrent pyogenic cholangitis) [7]. Chronic liver disease (cirrhosis and viral infection) is also considered a risk factor, especially for intrahepatic cholangiocarcinoma [8].

The primary treatment of cholangiocarcinomas is surgery [9]. The contribution of adjuvant and neoadjuvant therapy in resected patients is controversial. Meta-analysis has shown a survival contribution, especially in node-positive patients [10]. Adjuvant chemoradiotherapy is recommended in patients who do not undergo R0 resection or in node-positive patients [11]. In patients with unresectable/metastatic cholangiocarcinoma, the addition of immunotherapy (durvalumab or pembrolizumab) to gemcitabine plus cisplatin is recommended as the first step in patients with good Eastern Cooperative Oncology Group Performance Status (ECOG PS) and without hyperbilirubinemia. A contribution to the overall survival (OS) was observed in both studies in which durvalumab/pembrolizumab was added to chemotherapy [12,13]. The prognostic nutritional index (PNI) is a parameter calculated using albumin and absolute lymphocyte value, reflecting the immunological and nutritional status of the cancer patient. Recently, studies have been conducted showing the prognostic importance of PNI in many types of cancer such as esophageal cancer [14], colorectal cancer, and stomach cancer [15]. In clinical studies, patients' nutritional status and immunity have been found to be associated with tumor growth and prognosis [16]. Gallbladder and bile pathway tumors are very rare tumors, and the prognostic effectiveness of PNI has not been evaluated in this disease group.

The aim of our study is to evaluate the prognostic effectiveness of PNI in unresectable/metastatic gallbladder and biliary tract cancers.

## Materials and methods

Sixty-eight patients aged 18 years or over diagnosed with unresectable/metastatic gallbladder cancer and cholangiocarcinoma, between January 2014 and January 2023, were included in the study. The study was conducted in a single center. Patient files were examined retrospectively. Age, gender, ECOG performance status, date of diagnosis, etiological factors, first- and second-line treatment protocols, chemotherapy responses, albumin, bilirubin, complete blood count parameters at the time of diagnosis, final status, and final status dates were recorded.

Patients with missing data, unconfirmed diagnosis, curative surgery for early stage, and multiple primary tumors were excluded from the study.

The PNI was calculated using albumin and lymphocyte values (10 x albumin g/dL + 0.005 x total lymphocyte/mm^3^). The PNI of each patient at the time of diagnosis was calculated separately. The relationship between PNI and age, gender, ECOG PS, tumor localization, and CA19.9 level with OS was assessed.

OS was defined as the time from diagnosis to death or last visit for patients still alive. Survival data were last updated in March 2024. The primary endpoint of our study was to evaluate the relationship between OS and PNI.

Statistical analysis

All analyses were performed using the Statistical Product and Service Solutions (SPSS, version 23.0; IBM SPSS Statistics for Windows, Armonk, NY) program. In the descriptive statistics of the study, continuous variables were used as mean (standard deviation), and median (range); categorical variables were presented as frequency (percentage). The chi-square test or Fisher's exact test was used to compare the categorical variables of two independent groups. Independent sample t-test and Mann-Whitney-U test were used to compare parametric and non-parametric data, respectively. OS was estimated with the Kaplan-Meier method and compared with a log-rank test. The optimal cut-off value of PNI for predicting OS was determined with the receiver operating characteristic (ROC) curve. Univariate and multivariate logistic regression models were applied to evaluate the factors predicting OS. A logistic regression model was created with variables with a p-value of <0.05, and independent factors predicting OS were identified.

## Results

Sixty-eight patients were included in the study. The median age of the patients was 60 years, and 41 (60.3%) of the patients were female and 27 (39.7%) were male. Twenty-eight (41.2%) of the patients were diagnosed with gallbladder cancer, while 40 (58.8%) were diagnosed with cholangiocarcinoma (Table 1).

**Table 1:** Percentage of subgroups of a specific parameter should be given vertically (i.e., percentage of males and females in PNI < 44 group and >= 44 group) ECOG PS: Eastern Cooperative Oncology Group Performance Status, FOLFOX: 5-fluorouracil, oxaliplatin, leucovorin; PNI: Prognostic nutritional index

		PNI < 44 (N: 26)	PNI ≥ 44 (N: 42)	P value
Age (median) (min-max)		60 (95 % CI: 54-63)	62 (95 % CI: 56-63)	
Sex				
	Female	16 (39)	25 (61)	0.869
	Male	10 (37)	17 (63)	
Comorbidity				
	Yes	9 (28.1)	23 (71.9)	0.106
	No	17 (47.2)	19 (52.8)	
ECOG PS				
	0-1	12 (67.6)	34 (73.9)	0.003
	≥2	14 (63.6)	8 (36.4)	
Cholelithiasis History				
	Yes	3 (42.9)	4 (57.1)	0.790
	No	23 (37.7)	38 (62.3)	
Tumor Location				
	Gall bladder	13 (32.5)	15 (53.6)	0.245
	Cholangiocarcinoma	13 (46.4)	27 (67.5)	
Ca 19.9				
	<96	8 (26.6)	22 (73.4)	0.405
	≥ 96	11 (36.6)	19 (63.4)	
First Line Chemotherapy				
	No	4 (50.0)	4 (50.0)	0.499
	Gemcitabine+Cisplatin	12 (30.8)	27 (69.2)	
	Gemcitabine	4 (40.0)	6 (60)	
	Capecitabine	4 (57.1)	3 (42.9)	
	Gemcitabine+Oxaliplatin	1 (100)	0 (0)	
	Folfox	0 (0)	1 (100)	
	Carboplatin+Paclitaxel	1 (50.0)	1 (50.0)	
Second Line Chemotherapy				
	No			0.440
	Gemcitabine+Cisplatin	1 (20.0)	4 (80.0)	
	Gemcitabine	1 (16.7)	5 (83.3)	
	Capesitabine	1 (11.1)	8 (88.9)	
	Folfox	3 (42.9)	4 (57.1)	
	Gemcitabine+Nabpaclitaxel	0 (0)	1 (100)	
	Paclitaxel	0 (0)	1 (100)	

**Figure 1:**
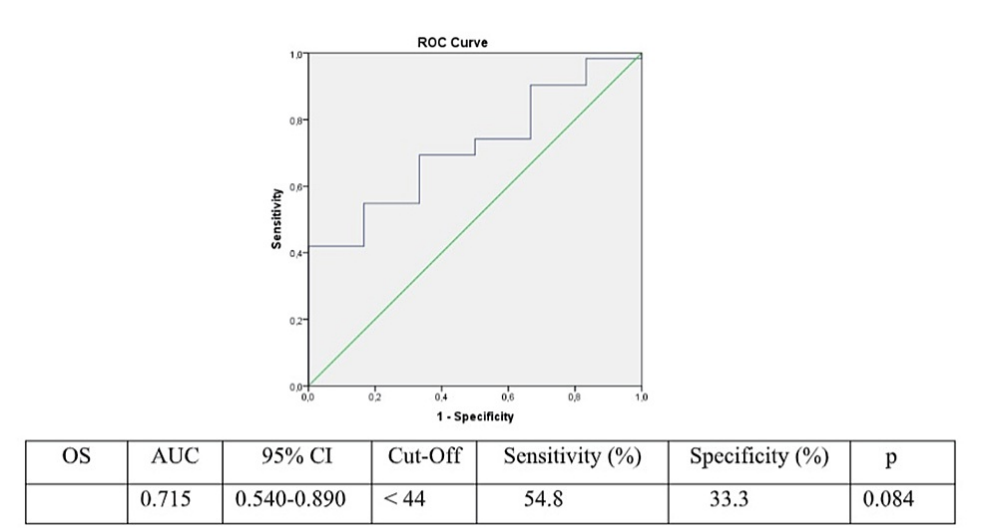
Value of prognostic nutritional index in predicting overall survival

The PNI mean of the patients included in the study was 44.8 (95% CI: 42.9-46.7), and the median was 44.77 (minimum: 22, maximum: 61.4). ROC analysis demonstrated a statistically significant prediction of OS when the PNI was < 44. (AUC: 0.715, sensitivity: 54.8%, specificity: 33.3%; p=0.08) (Figure 1).

**Figure 2:**
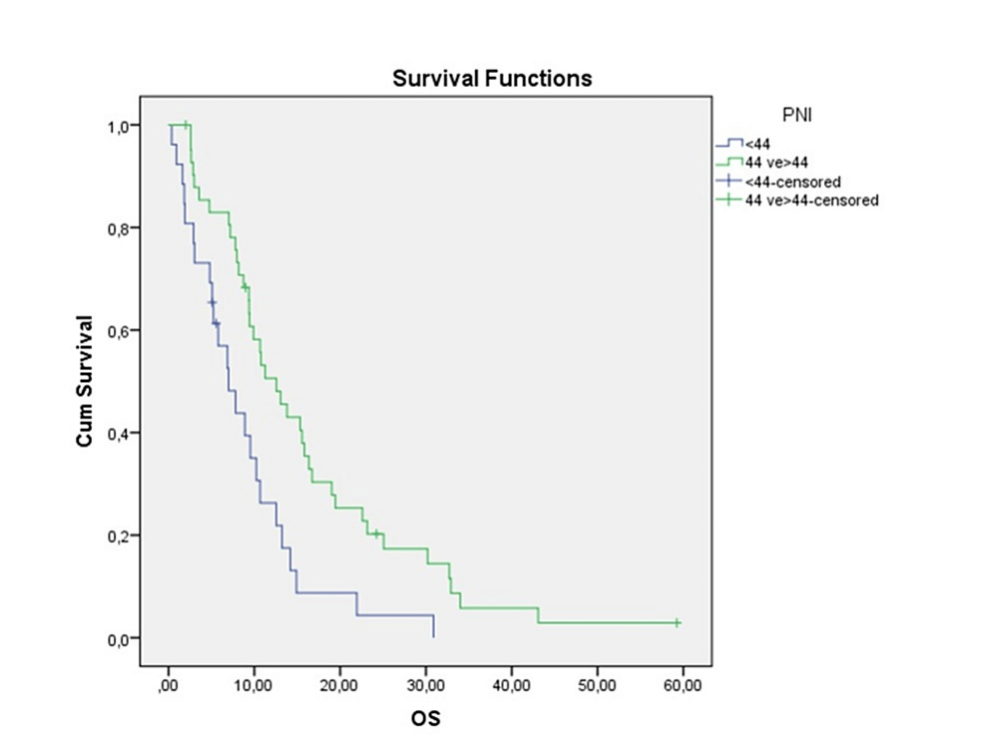
Kaplan-Meier curve according to the prognostic nutritional index OS: Overall survival, PNI: Prognostic nutritional index

Patients were divided into two groups: PNI < 44 and ≥ 44. The median OS in the PNI < 44 group was 6.9 months (95% CI: 3.92-10.06). In the PNI ≥ 44 group, the median OS was 12.58 months (95% CI: 8.76-16.39). OS was statistically significant between the two groups (p = 0.004) (Figure 2).

Sixty of our patients were able to receive chemotherapy. The prognostic effectiveness of PNI was evaluated in patients receiving chemotherapy. In patients receiving chemotherapy, the median survival was found to be 8.93 months in the PNI < 44 group, while the median survival was found to be 12.58 months in the PNI ≥ 44 group. The difference between both groups was statistically significant (p = 0.010) (Table 2).

**Table 2:** Median survival according to PNI groups in patients receiving chemotherapy PNI: Prognostic nutritional index

	PNI < 44 (months)	PNI ≥ 44 (months)	P value
Receiving Chemotherapy	8.90	12.58	0.010

The median value of Ca 19.9 was 96. When the patients were divided into two groups according to the median value, the median OS was 14.1 months in the < 96 group, while the mean OS was 7.9 months in the ≥ 96 group. OS was statistically significant between the two groups (p=0.005) (Table 3).

**Table 3:** Univariate and multivariate analyses of factors related to overall survival HR: Hazard ratio, ECOG PS: Eastern Cooperative Oncology Group Performance Status, Ca 19.9: Cancer antigen 19.9, PNI: Prognostic nutritional index

Factor	Univariate Analysis		Multivariate Analysis	
	95 % CI	p	HR (95 % CI)	p
Age(<65 yaş vs ≥65 yaş)	0.73-2.09	0.41		
Sex	0.58-1.61	0.90		
ECOG PS	1.97-6.59	0.00	1.76-6.03	0.00
Tumor Location	0.59-1.34	0.96		
Ca 19.9	0.25-0.79	0.005	0.24-0.77	0.004
PNI	0.26-0.76	0.004	0.29-0.90	0.02

In univariate analysis, the ECOG performance status, Ca 19.9, and PNI were statistically significant variables in predicting OS (p < 0.05). In multivariate analysis, the ECOG performance status, Ca 19.9, and PNI were found to be independent factors in predicting OS (p < 0.05) (Table 3).

## Discussion

In our study, we found that the PNI is prognostic in terms of OS in patients with unresectable/metastatic gallbladder cancer and cholangiocarcinoma and could be informative to the clinician in patient follow-up. There are many studies evaluating the prognostic effectiveness of PNI in cancer patients. Sun et al. included 14 studies in their meta-analysis, and it was shown that PNI was associated with OS and postoperative complications in cancer patients [17]. In another study, including 3,393 gastric cancer patients, it was found that low PNI was an indicator of advanced TNM stage and was associated with lower OS [15]. Studies have been conducted on the prognostic effectiveness of PNI in other types of cancer, as well as gastrointestinal system tumors. A meta-analysis of lung cancer patients showed that low PNI value was associated with shorter PFS and OS in both non-small cell lung cancer (NSCLC) and small cell lung cancer (SCLC) patients. Being overweight is common in breast cancer patients, as opposed to malnutrition [18]. In a study evaluating the relationship of PNI with pathological response and survival in breast cancer, it was found that higher PNI values were associated with lower pathological complete response and shorter disease-free survival [19].

Gallbladder cancer and cholangiocarcinoma are rare tumors of the gastrointestinal system, data in this area are limited, and various studies are needed in both diagnosis and treatment methods. The most important prognostic factor in gallbladder tumors is the disease stage. Studies have found that age, gender, and surgery methods are also prognostic [20]. In a study conducted by Wang et al., a nomogram was developed to calculate the benefit of adjuvant chemotherapy and chemoradiotherapy in operated gallbladder cancers. In this study, it was determined that ≥ T2 and ≥ N1 tumors had the greatest benefit from adjuvant chemoradiation [21]. In another study evaluating prognostic factors in metastatic gallbladder cancer, neutrophil/lymphocyte ratio, Ca 19.9 level, carcinoembryonic antigen (CEA) level, and the presence of liver metastasis were found to be prognostic [22]. In our study, the Ca 19.9 level was found to be prognostic, consistent with this study.

Cholangiocarcinoma is a heterogeneous group, including intrahepatic and extrahepatic tumors, and prognostic factors were evaluated separately in subgroups. The most important prognostic factor in both groups is the disease stage, as in gallbladder tumors. The prognosis of resectable tumors is significantly better. A study found that, among the components of the American Joint Committee on Cancer (AJCC) staging, nodal positivity affects staging most strongly. In this study, microvascular invasion was found to be prognostic, as well as the AJCC stage [23]. In a study conducted on metastatic cholangiocarcinoma, the Ca 19.9 level and ECOG performance status were found to be prognostic [24]. In our study, both factors were found to be prognostic, consistent with this study.

In our study, we evaluated the prognostic effectiveness of PNI in the subgroup of patients who could receive chemotherapy. Patients who could not receive chemotherapy were generally unable to receive treatment because their performance was poor or their laboratory parameters were not suitable for chemotherapy. The low PNI in this group was the result we expected. Patients who could receive chemotherapy constituted a more homogeneous group. For this reason, we think that the prognostic importance of PNI may be more valuable in this group.

## Conclusions

In conclusion, in our study, we evaluated the prognostic effectiveness of the PNI in two rare tumors and found that it was associated with survival. It is very valuable as it is the first study in the literature to evaluate the effectiveness of the PNI in these tumors. We believe that the PNI can be used as a marker to assist the clinician in evaluating the prognosis of patients in the clinic and predicting treatment tolerance. The main limitations of our study were the small number of patients and the retrospective study design.
